# Estimating Marginal Odds Ratios for Cluster Randomized Trials: The Cluster‐Level GLM

**DOI:** 10.1002/sim.70794

**Published:** 2026-10-21

**Authors:** Jennifer A. Thompson, Brennan C. Kahan, Andrew Copas

**Affiliations:** ^1^ Department of Infectious Disease Epidemiology and International Health London School of Hygiene and Tropical Medicine London UK; ^2^ Medical Research Council Centre of Research Excellence in Clinical Trial Innovation in Partnership With NIHR University College London London UK

**Keywords:** cluster level analysis, cluster randomized trial, generalized linear model, informative cluster size, marginal odds ratio, simulation study

## Abstract

**Background:**

Methods for estimating marginal effects in cluster randomized trials are limited. We describe and evaluate a cluster‐level generalized linear model (CL‐GLM) that applies a GLM to cluster‐level summaries (means, proportions, or rates) and can estimate differences, ratios, or odds ratios. We focus on odds ratios.

**Methods:**

We conducted a simulation study with 10–50 clusters, intracluster correlation coefficients of 0.05 or 0.15, and a mean cluster size of 120, either fixed or variable. Informative cluster size, where the intervention effect varies with cluster size, was also considered. Control arm prevalence was set to 50%. We compared cluster‐level GLMs with normal and gamma distributions and logit link to generalized estimating equations (GEE) and independence estimating equations (IEEs). Weighted and unweighted analyses were examined to target cluster‐average and individual‐average estimands.

**Results:**

CL‐GLM with a normal distribution produced unbiased marginal cluster‐average odds ratios with appropriate confidence interval coverage. CL‐GLM with a gamma distribution resulted in over‐coverage. Unweighted IEE produced unbiased marginal individual‐average odds ratios with correct coverage. GEE was unbiased when cluster size was uninformative but biased under informative cluster size. Weighted CL‐GLM led to under‐coverage, whereas weighted IEE resulted in over‐coverage. The CL‐GLM had similar power to GEE and unweighted IEE.

**Conclusions:**

The cluster‐level GLM with a normal distribution provides a robust alternative to estimating equation approaches for marginal odds ratios in cluster randomized trials, with good performance under both informative and uninformative cluster size and no loss of power. We recommend the cluster‐level GLM with a normal distribution when the marginal cluster‐average estimand is of interest, and unweighted IEEs when the marginal individual‐average estimand is of interest.

## 1 Background

The recent focus on estimands, a precise definition of the intervention effect, in clinical trials has led to a better understanding of estimands in cluster randomized trials (CRTs) [1]. In CRTs, hospitals, communities, or other ‘clusters’ of individuals are randomized to an intervention or control condition. Individuals in the same cluster are usually more similar to one another than individuals in different clusters, so their outcomes are correlated. One unique aspect of an estimand for CRTs is the choice of a marginal or cluster‐specific intervention effect [2]. Marginal estimands compare contrasts of population summaries, and might be of more interest to population decision makers such as policy makers and to individuals that can move clusters. Cluster‐specific estimands compare contrast within each cluster first and then average these over clusters. These might be of more interest to cluster decision makers, such as hospital managers, and when an individual is fixed in their cluster [3]. For many types of intervention effect such as mean differences and risk differences, these will coincide with one another, but for non‐collapsible intervention effects such as odds ratios they will differ when there is correlation between individuals within clusters and the difference will depend on the strength of that correlation [2]. Different analysis methods will target different estimands.

The analysis of a CRT must account for the correlation of individuals within clusters, and generalized estimating equations with an exchangeable correlation structure (hereafter “GEE”) and independence estimating equations (IEEs) are the most commonly used approaches that estimate marginal effects. However, CRTs tend to have a small number of clusters, and these approaches require careful specification with small sample corrections to estimate confidence intervals (CIs) the correct width (coverage) and *p* values the correct size (Type I Error). There is an existing cluster‐level analysis, a cluster‐level *t*‐test [4], which does not require these small sample corrections. However, this method targets a cluster‐specific intervention effect, although there has been some confusion over this in past literature [3, 5].

Research has identified another aspect of estimands in CRTs with varying cluster size related to how individuals are weighted in the estimands definition. Different intervention effects can be targeted by either giving equal weight to individuals or equal weight to clusters. Giving equal weight to clusters gives a cluster‐average effect, and giving equal weight to individuals within clusters gives an individual‐average effect. When the cluster size is related to the outcome values under control or to the effect of treatment (informative cluster size) these effects differ; otherwise they coincide [6]. IEE target marginal individual‐average effects and GEE target neither. Although methods have been proposed for marginal cluster‐average effects [3] none have been validated in simulation studies.

In this article, we describe the novel cluster‐level generalized linear model (GLM) analysis method for estimating marginal treatment effect in CRTs. It targets a marginal cluster‐average treatment effect, and is unbiased whether or not cluster size is informative [3]. The method was first presented at the International Society of Clinical Biostatistics in 2023 [7], and subsequently described by Kahan et al. [3]. The present paper provides the first full description and empirical validation of the method.

In the next section, we first define the four possible estimands of interest. We then present a simulation study to validate the cluster‐level GLM and compare it to other analysis approaches that estimate marginal effects, under both uninformative and informative cluster size. We focus on estimating an odds ratio, as this is commonly used and highlights the difference between the estimands. We demonstrate these methods with a motivating example before providing guidance to those conducting a CRT.

## 2 Estimands

CRTs can estimate four different estimands, depending on which combination of marginal vs. cluster‐specific and individual‐ vs. cluster‐average are used: (i) marginal individual‐average; (ii) marginal cluster‐average; (iii) cluster‐specific individual‐average; and (iv) cluster‐specific cluster‐average. Formal definitions of these estimands have been given previously [3] and we provide a brief overview here.

Marginal estimands compare contrasts of population‐summaries, that is, they compare the mean (or odds) of all individuals under the intervention vs. the mean (or odds) of all individuals under the control. Conversely, cluster‐specific estimands compare the mean (or log odds) within each cluster first, and then average over these cluster‐specific effects to obtain an overall “cluster‐specific” estimand. For differences in means or proportions, values of the marginal and cluster‐specific estimands will be the same, however for odds ratios they will differ when the intracluster correlation coefficient (ICC) is nonzero with larger ICC values leading to larger differences.

Individual‐average estimands give each individual equal weight; in contrast, cluster‐average estimands give each cluster equal weight, so individuals belonging to larger clusters will have smaller weight in the estimand than individuals belonging to smaller clusters. The value of these two estimands will coincide unless there is informative cluster size, that is, if the outcome or treatment effects differ in smaller clusters compared to larger clusters. For a difference of means or proportions, the individual‐average and cluster‐average estimands will only differ if the treatment effect differs by cluster size. For odds ratio, they will differ if either the outcome or treatment effect differs by cluster size.

## 3 Analysis Methods

Analysis methods for CRTs must account for the correlation of observations within clusters and work with a small number of clusters as most CRTs have fewer than 40 clusters [8]. Below, we provide details of analysis methods that target each of the marginal odds ratio estimands. All analysis methods compare test statistics to a t‐distribution with the number of clusters minus two as the degrees of freedom.

### 3.1 Marginal Cluster‐Average Odds Ratio

#### 3.1.1 Cluster‐Level GLM

In this paper, we provide a detailed description of the novel cluster‐level GLM analysis method, which will target a marginal cluster‐average odds ratio. First, the proportion of events is calculated within each cluster (denoted as $p_{i}$). These are then used as the outcome of a GLM with a logit link function to estimate an odds ratio. The distribution family for the GLM must have a variance that is independent of the mean, for instance the normal distribution or gamma distribution, so that the model can incorporate the between cluster variability.

We denote these approaches as the *unweighted cluster‐level GLM(normal)* method (*unCL‐GLM(normal)*), and the *unweighted cluster‐level GLM(gamma)* method (*unCL‐GLM(gamma)*). For ratio estimands, a log link can be used, and for a difference, the identity link. Details of how to implement this in the statistical software Stata are provided in the Supplementary Text 1.

#### 3.1.2 Weighted Independence Estimating Equations (wIEEs)

Another approach to estimating a marginal cluster‐average odds ratio is to use wIEE. IEE analyze the individual level outcomes using a logistic regression that assumes independence between all individuals in conjunction with cluster‐robust standard errors. This can be implemented using GEE with an independence working correlation structure or a maximum‐likelihood based logistic regression model. Cluster‐robust standard errors must be used to account for correlation, and small sample corrections are required in most cases. We use the Fay and Graubard small sample correction, which has performed well for CRTs previously [9]. To target the marginal cluster‐average odds ratios, participants are weighted by the inverse of their cluster size. We denote this as *wIEE*. To our knowledge, there is no research into whether this weighted approach provides unbiased standard errors.

### 3.2 Marginal Individual‐Average Odds Ratios

#### 3.2.1 Weighted Cluster‐Level GLM

The cluster‐level GLM analysis can be modified to estimate a marginal individual‐average estimand by weighting the cluster‐level proportions by the number of participants in each cluster. We denote this approach as the *weighted cluster‐level GLM(normal)* (*wCL‐GLM(normal)*), and similarly the *weighted cluster‐level GLM(gamma)* (*wCL‐GLM(gamma)*) with a normal and gamma distribution respectively. These weighted cluster‐level GLMs use robust (Huber–White) standard errors.

#### 3.2.2 IEEs

IEEs without weights can also be used to estimate a marginal individual‐average odds ratio. We refer to this approach as *unIEE* to denote no weighting is used. There are many published simulation studies showing that unIEE provides unbiased estimates of the marginal odds ratio and CIs of the correct width when cluster size is not informative [9, 10].

### 3.3 Other Marginal Odds Ratio Analysis Methods

GEEs are another common approach to estimating marginal odds ratios. These use a binomial family and logit link and specify a working correlation structure for the correlation within clusters in conjunction with cluster‐robust standard errors with a small sample correction. Most often an “exchangeable” working correlation structure is used, which we denote here as *unGEE*. This approach estimates a marginal odds ratio. GEEs with an exchangeable correlation structure are unbiased in the absence of informative cluster size, but in the presence of informative cluster size, they do not target the cluster‐average or individual‐average treatment effect [3].

### 3.4 Cluster‐Specific Odds Ratio

While this paper focuses on marginal odds ratios, we provide a brief overview of the alternative cluster‐specific odds ratio analysis.

A well‐known approach to cluster‐level analysis is a cluster‐level *t*‐test. This targets a cluster‐specific cluster‐average odds ratio [3]. First, one calculates the proportion of events within each cluster ($p_{i}$). This is then transformed to a log odds for each cluster ($\log\left(odds_{i}\right)=\log\left(p_{i}/\left\{1-p_{i}\right\}\right)$). Then, a *t*‐test or linear regression model is applied to each cluster‐level log‐odds. We denote this approach as the *unweighted cluster‐level t*‐test *(unCL‐TTEST)*. There are many published simulation studies demonstrating that this is a robust approach when cluster size is not informative [5, 9]. It is a limitation of this approach that it is not straightforward to include clusters in which no outcome events are observed as the log(odds) cannot be calculated for these clusters. Usually, an ad‐hoc corrections is used to allow their inclusion such as adding half an event and half a non‐event to each cluster. Note that this is not an issue for the cluster‐level GLM method. The above approach can be modified to estimate the cluster‐specific individual‐average odds ratio using weights. Since we focus on marginal odds ratios, this method is not considered further.

Mixed effect logistic regression is another commonly used analysis method for CRTs. This approach estimates a cluster‐specific odds ratio, so it is not considered further.

## 4 Simulation Study Methods

To explore the accuracy and efficiency of these analysis methods, we conducted a simulation study. All code to replicate this simulation study is available at: https://github.com/jenniferthompson1/Cluster_level_GLM.

We had three aims:

1. Empirically demonstrate that the cluster‐level GLM method targets the marginal odds ratio.
2. Compare the cluster‐level GLM approach to other analysis methods that target the marginal odds ratio under uninformative cluster‐size.
3. Compare marginal odds ratio analysis approaches under informative cluster size.

### 4.1 Data Generating Mechanism

The data generation approach was the same for all three aims. Data were simulated from CRTs with an equal allocation of clusters to a control and an intervention condition to test whether an intervention would reduce the prevalence of a binary outcome. Trials had a total of either 10, 20, or 50 clusters. The cluster‐level prevalences of the outcome $Y_{j}$ were generated using the following model:

$$logit\left(Y_{j}\right)=\beta_{0}+\beta_{1}x_{j}+u_{j}$$

where *j* indicated the cluster, $x_{j}$ indicates trial arm (1 for clusters allocated to the intervention and 0 for clusters allocated to the control), and $\beta_{0}$ is the cluster‐specific log odds of the outcome in the control condition. $\beta_{0}$ was 0 for all simulations, equivalent to a prevalence of 0.5. $\beta_{1}$ was the log odds ratio intervention effect. In the simulations with uninformative cluster size (Aims 1 and 2), $e^{\beta_{1}}=0.75$. In simulations with informative cluster size (aim 3), $e^{\beta_{1}}=0.75$ for clusters with more than 120 observations and $e^{\beta_{1}}=1$ otherwise. $u_{j}˜N\left(0,\sigma \right)$ is the cluster mean variability. σ was determined by the ICC using $\mathrm{ICC}=\sigma^{2}/\left(\sigma^{2}+\frac{\pi^{2}}{3}\right)$ [11]. The ICC was either 0.05, which is a common value for a health outcome, or a higher value that still happens in practice of 0.15 [12].

Individual level outcomes were then sampled from a binomial distribution with their cluster's prevalence. We simulated data where all clusters had 120 observations, and where clusters varied in size with a mean of 120 observations and a coefficient of variation of cluster size of 0.5 sampled from a negative binomial distribution [9]: This results in 50% of clusters with a cluster size between 76 and 153 observations, and 90% of clusters between 41 and 233 observations.

We simulated 3000 trials for all combinations of each parameter that we varied. Table 1 shows a summary of the scenarios.

**TABLE 1:** Summary of the simulation study scenarios.

Characteristic	Values
*Simulation Study 1 (Aims 1 and 2)*	
Total number of clusters	10, 20, 50
Mean cluster size	120
Coefficient of variation of cluster size (CV)	0, 0.5
Control‐arm prevalence	0.5
ICC	0.05, 0.15
Intervention effect	Cluster‐specific OR = 0.75 same for all clusters
*Simulation Study 2 (Aim 3)*	
Total number of clusters	10, 20, 50
Mean cluster size	120
Coefficient of variation of cluster size	0.5
Control‐arm prevalence	0.5
ICC	0.05, 0.15
Intervention effect	Cluster‐specific OR = 0.75 if clusters had more than 120 observations, OR = 1 otherwise

To determine the true marginal cluster‐average and marginal individual‐average odds ratios for each scenario, we simulated a CRT with 100 000 clusters to calculate each effect. Further details are given in Supplementary Methods Text 2.

### 4.2 Estimands

To address aims one and two (without informative cluster size), we estimated marginal odds ratios. Aim one also required estimation of the cluster‐specific odds ratio. Note that individual‐average and cluster‐average effects coincide here due to uninformative cluster size. For aim three (with informative cluster size), we estimated marginal cluster‐average odds ratios and marginal individual‐average odds ratios.

### 4.3 Analysis Methods

#### 4.3.1 Aim 1: Demonstrate That the Unweighted Cluster‐Level GLM Targets the Marginal Odds Ratio

For Aim 1, we analyzed simulated data with the cluster‐level GLM with a normal distribution (CL‐GLM(normal)) and a gamma distribution (CL‐GLM(gamma)), and the cluster‐level *t*‐test analysis (unCL‐TTEST) to demonstrate that these analysis approaches target different odds ratios.

#### 4.3.2 Aim 2: Comparison of Marginal Odds Ratio Analysis Methods Under Uninformative Cluster Size

To address aim two, we analyzed simulated data with analysis methods that target marginal odds ratios: the cluster‐level GLM with a normal distribution (unCL‐GLM(normal)) and a gamma distribution (unCL‐GLM(gamma)), wIEE, weighted cluster‐level GLM with a normal distribution (wCL‐GLM(normal)), unweighted independence estimating equations (unIEE), and unweighted generalized estimating equations with an exchangeable working correlation matrix (unGEE).

#### 4.3.3 Aim 3: Comparison of Marginal Odds Ratio Analysis Methods Under Informative Cluster Size

We analyzed data with unCL‐GLM(normal) and wIEE to estimate the marginal cluster‐average odds ratio. unCL‐GLM(gamma) was not used due to inferior performance in aim 2. To estimate the marginal individual‐average odds ratio, we analyzed data with wCL‐GLM(normal) and unIEE. unGEE was also included to explore its performance despite not targeting either estimand.

### 4.4 Performance Evaluation

We calculated the log odds ratio and its 95% CI for the estimated intervention effect from each analysis method and each scenario.

We estimated bias, relative error in standard errors (reported in Supplementary Tables S1 and 6), 95% CI coverage, and power. Each method was assessed against the true odds ratio that the method was targeting or for unGEE we used the marginal individual‐average effect as the true target is unclear. CI coverage calculated the proportion of CIs that contained the odds ratio that the method was targeting. Each measure is reported along with a 99% CI: 99% intervals were used to reduce the Type I Error.

Data generation and all cluster‐level analyses were conducted in Stata version 17, and performance evaluation used the Stata command simsum [13]. GEEs and IEEs were conducted in R v4.3.1 since small sample corrected methods for GEE in Stata do not allow weights.

## 5 Simulation Study Results

Convergence was very good throughout the simulation study: only four GEE models failed to converge in the scenario with 10 clusters, a low ICC, and variable cluster size.

### 5.1 Aim 1: Demonstrate That the Unweighted Cluster‐Level GLM Targets the Marginal Cluster‐Average Odds Ratio

Figure 1 shows the mean estimated odds ratios for each scenario with uninformative cluster size for the three cluster‐level analysis methods. The cluster‐level GLM methods provided identical, unbiased estimates of the marginal odds ratio within 0.009 of the true odds ratio. The cluster‐level *t*‐test method provided unbiased estimates of the cluster‐specific odds ratio always within 0.031 of the true odds ratio.

**FIGURE 1:**
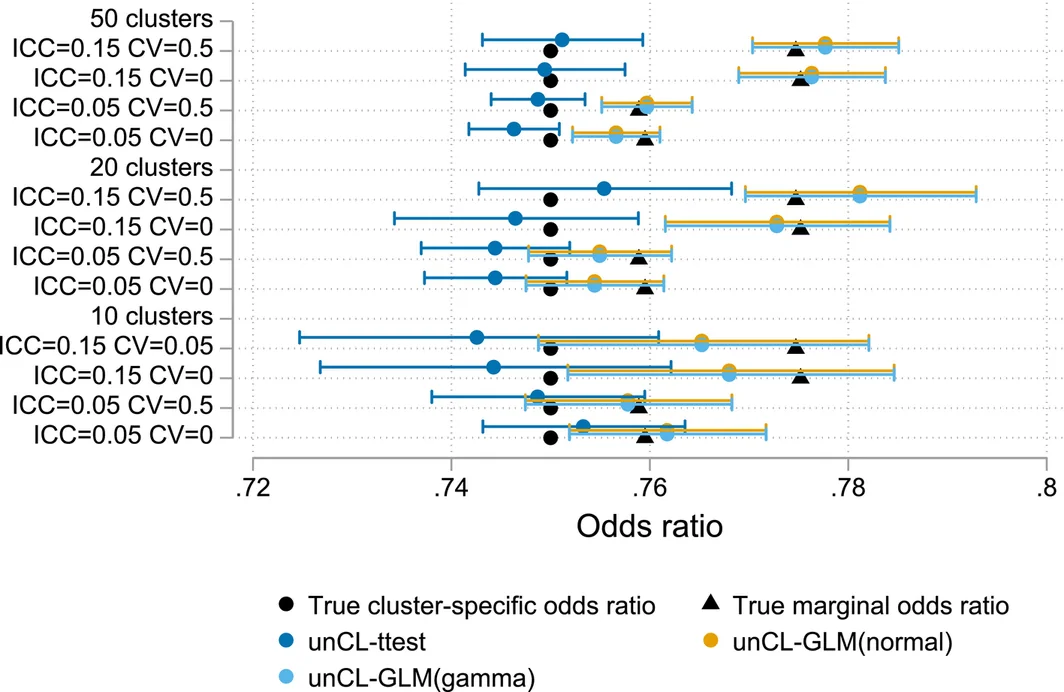
Mean intervention effect odds ratio estimates and 99% confidence intervals for scenarios with uninformative cluster size.

### 5.2 Aim 2: Comparison of Marginal Odds Ratio Analysis Methods Under Uninformative Cluster Size

All methods provided unbiased estimates of the marginal odds ratio with uninformative cluster size (Supplementary Table 1).

The cluster‐level GLM with a normal distribution (unCL‐GLM(normal)) gave close to 95% coverage in all scenarios with coverage ranging from 94.5% to 95.8% (Figure 2). With 10 or 20 clusters, the cluster‐level GLM with a gamma distribution (unCL‐GLM(gamma)) had CI coverage larger than 95% (96.1% to 96.4%) with an ICC = 0.15, but good coverage with 50 clusters or 10 or 20 clusters with ICC = 0.05.

**FIGURE 2:**
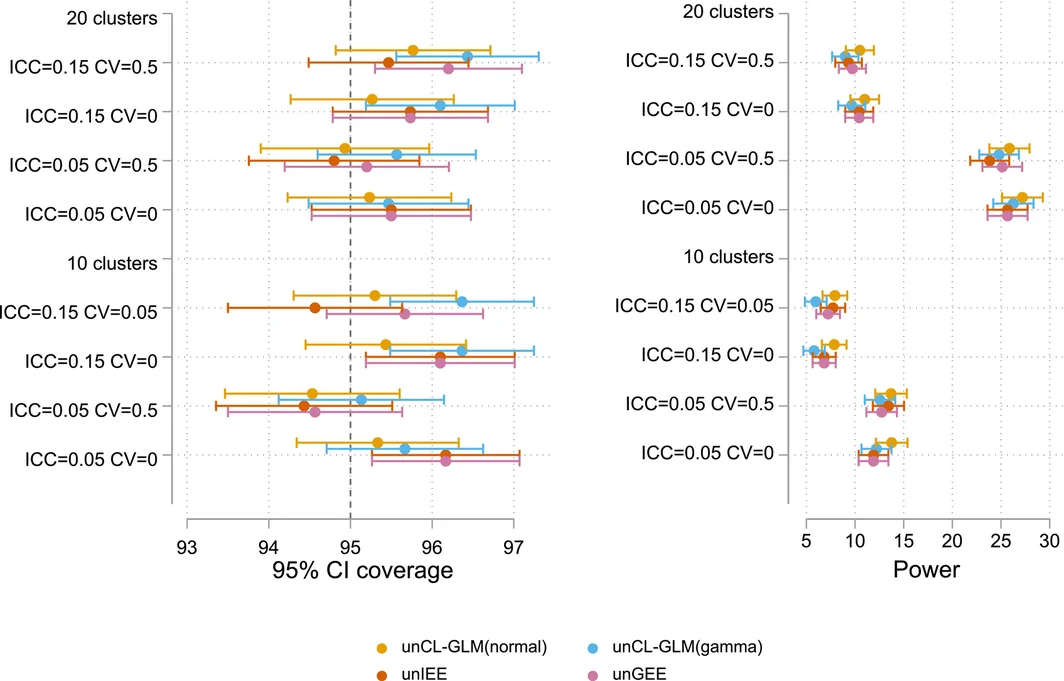
Confidence interval coverage and power for scenarios with uninformative cluster size and 10 or 20 clusters.

With a common cluster size, IEE (unIEE) and GEE (unGEE) are identical methods. These have CI coverage close to 95% with 20 or 50 clusters (94.7% to 95.7%) but slightly high coverage with 10 clusters (96.1% and 96.2% with low and high ICC) (Figure 2 and Supplementary Table 3). With varying cluster size, unIEE and unGEE also had generally good CI coverage (94.4%–96.2%).

Weighted cluster‐level GLM with a normal distribution (wCL‐GLM(normal)) tended to have low CI coverage with 20 or fewer clusters when cluster size varied (91.2% and 91.7% with 10 clusters and varying cluster size for low and high ICC, respectively, Supplementary Table 3), but close to nominal coverage with 50 clusters (93.9% to 95%). With common cluster size, wIEE was identical to the unweighted methods, but with varying cluster size, it had high CI coverage (96.2%–97.3%).

All analysis methods with coverage close to 95% had power within 5% of one another in each scenario (Supplementary Table 4). Of note, the power of unCL‐GLM(normal) was always larger than unGEE with a mean 0.8% difference.

### 5.3 Aim 3: Comparison of Marginal Odds Ratio Analysis Methods Under Informative Cluster Size

Figure 3 shows the mean estimated odds ratios under informative cluster size. wCL‐GLM(normal) and unIEE gave identical, unbiased estimates of the marginal individual‐average odds ratio. unCL‐GLM(normal) and wIEE gave identical, unbiased estimates of the marginal cluster‐average odds ratio. unGEE did not target either of these estimands: unGEE estimated odds ratios between 0.92 and 0.93 where true values ranged from 0.89 to 0.9 for the cluster‐average odds ratio and 0.84 and 0.85 for the individual‐average odds ratio.

**FIGURE 3:**
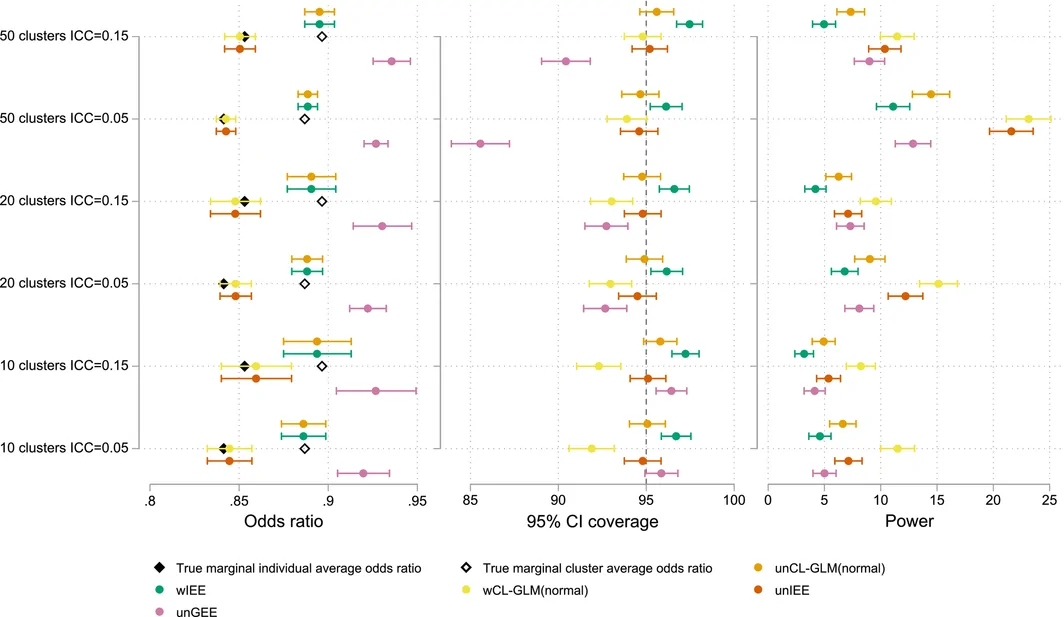
Mean odds ratio estimates, 95% confidence interval coverage and power with informative cluster size.

Comparing marginal cluster‐average methods, the cluster level GLM with a normal distribution (unCL‐GLM(normal)) gave CIs with close to 95% coverage, but wIEE gave CIs with coverage over 95% (96.1% –97.5%). This gave unCL‐GLM(normal) greater power than wIEE (between 1.7% and 3.4% more power).

Comparing marginal individual‐average methods, wCL‐GLM(normal) gave CIs that were too narrow particularly with a small number of clusters: with 10–20 clusters, coverage was between 91.9% and 93%. unIEE gave CIs with close to 95% coverage. Since coverage was low for wCL‐GLM(normal), we did not compare power.

Comparing unGEE to the marginal individual‐average effect, CIs did not have correct coverage owing to its bias for this estimand.

Comparing power across estimands, unCL‐GLM(normal) estimating the marginal cluster‐average effect had between 0.5% and 5.9% lower power than unIEE estimating the marginal individual‐average effect.

## 6 Motivating Example

Tuberculosis treatment involves daily or every other day treatment for 6 months [14], and it is difficult for patients to maintain good adherence to treatment throughout such a long treatment period. New medication monitor boxes used to store medication can provide patients with a sound and a light to remind them to take their medication, and the box can feed information on adherence (based on box openings) back to their clinician. Text messages to remind patients to take their medication have also been proposed as an approach to improve adherence.

In 2011, a large CRT randomized 36 counties in China to provide patients diagnosed with TB with standard of care, a medication monitor box, text messages, or both the medication monitor box and text messages. Here, we will focus on the 18 counties comparing standard or care and the medication monitor box. The primary outcome of the trial was months of treatment with at least 20% of doses missed, and there was strong evidence that the medication monitor box reduced months of treatment with more than 20% of doses missed (*p* = 0.002). Here, we focus on a simpler secondary outcome of patients that missed at least 10% of doses. This outcome had an ICC of 0.07 and was available for 1091 patients in the standard of care arm and 992 patients in the medication monitor box arm.

The mean cluster size was 116 with a coefficient of variation of cluster size of 0.1 ranging from 84 to 133 patients per cluster. There was weak evidence (*p* = 0.08) that the risk of a patient missing 10% of doses was lower in larger clusters with the chance of a patient missing 10% of doses reducing by 5% with every 10 extra patients in a cluster.

Table 2 shows the results for this trial using each of the analysis methods from our simulation study. The unweighted cluster‐level GLM approaches estimated odds ratios of 0.48, and the IEE and weighted cluster‐level GLM methods both give an estimated marginal population‐average odds ratio of 0.46. Here, the individual‐average and cluster‐average effects have only a small difference. The cluster‐level *t*‐test provides a slightly larger cluster‐specific odds ratio estimate (0.45 95% CI, 0.25, 0.83). All methods give a similar level of evidence for the intervention effect (*p* values between 0.01 and 0.02) and CIs of a similar width except the weighted cluster‐level GLM, which provides slightly stronger evidence (*p* = 0.007) and a narrower CI (0.26–0.78): this is likely due to the low CI coverage of this approach observed in the simulation study.

**TABLE 2:** Motivating example results.

Target estimand	Analysis method	Odds ratio (95% confidence interval)	*p*
Marginal cluster‐average	unCL‐GLM (normal)	0.48 (0.27, 0.85)	0.015
	unCL‐GLM (gamma)	0.48 (0.26, 0.88)	0.021
	WIEE	0.48 (0.27, 0.85)	0.015
Marginal individual‐average	unIEE	0.46 (0.26, 0.81)	0.010
	wCL‐GLM (normal)	0.46 (0.26, 0.78)	0.007
Marginal	unGEE	0.48 (0.26, 0.85)	0.016
Cluster‐specific cluster‐average	unCL‐ttest	0.45 (0.25, 0.83)	0.013

## 7 Discussion

In this paper, we have validated the cluster‐level GLM, a novel method of analysis for CRTs, and studied the properties of this approach for analyzing binary outcomes. We show that this approach targets a marginal odds ratio unlike the cluster‐level *t*‐test, and we found that specifying a normal distribution for the GLM lead to better CI coverage than specifying a gamma distribution. Performance was good even with as few as 10 clusters. With uninformative cluster size, we found comparable power using this approach and individual level analysis methods that also target the marginal odds ratio. With informative cluster size, we demonstrate that the unweighted cluster‐level GLM targets the cluster‐average odds ratio, has good CI coverage, and higher power than weighted independent estimating equations. We also considered a weighted cluster‐level GLM to estimate an individual‐average odds ratio, however this gave CIs that were too narrow.

We have shown that the cluster‐level GLM had comparable power compared to methods estimating the same estimand across our simulations including variable cluster size as well as demonstrating correct CI coverage. There are no other studies of the cluster‐level GLM, but it is often believed that cluster‐level analysis will have lower power than other individual‐level analysis methods [4, 15]. However, Austin did not apply appropriate small sample corrections to the individual‐level analysis methods, so biased standard errors mean the comparison of power is not appropriate [15], and Hayes and Moulton were only considering a large number of clusters [4]. We found that methods estimating the marginal cluster‐average odds ratio had lower power than methods estimating a marginal individual‐average odds ratio, but this is likely because the cluster‐average odds ratio was smaller in our simulations.

Where informative cluster size is of concern, we recommend using an unweighted analysis that targets the desired estimand, that is the cluster‐level GLM for a cluster‐average effect and IEE for a participant average effect, since standard errors were often biased for weighted analysis. Researchers may want to exercise caution and consider this approach even if unsure whether informative cluster size may arise.

The cluster‐level GLM performed well with both a large and small number of clusters. We found comparable performance to estimating equations with small sample corrections applied to the standard error and degrees of freedom with uninformative cluster size. Others have also found that the estimating equations can be applied with a small number of clusters as long as the standard errors use a small sample correction and a degree of freedom correction is applied [9, 10].

With informative cluster size, only unweighted approaches provided CIs with coverage close to 95%, so the choice of approach should depend on the odds ratio researchers wish to target. Consistent with other simulations for weighted analysis of CRTs [9, 16], we found that standard errors of the weighted cluster‐level GLM were too small, despite using a robust standard error, due to the small number of clusters. Standard errors for the wIEEs were too large. Further research is needed to develop approaches that estimate appropriate standard errors with weighted analysis methods or assess other analysis approaches such as bootstraps.

## 8 Strengths and Limitations

We have conducted a broad simulation study with a large number of repetitions for each scenario and scenarios chosen to reflect common situations in practice. However, there are many scenarios that have not been covered by our simulations. We have also only considered a common outcome, and the normal distribution may not be appropriate with rarer outcomes where the cluster log‐odds are likely to be more skewed. We used a simple data generating mechanism to create informative cluster size that is unlikely to occur in practice; however, we think this is sufficient to demonstrate the performance of the methods. The cluster‐level GLM can be used with different outcome types and to estimate risk ratios or risk differences, and future work is needed to assess its performance in these areas: note that for the risk difference, the cluster‐level GLM and cluster‐level *t*‐test are equivalent. Future work could look at alternative standard error estimators and non‐parametric approaches for weighted analysis methods.

## 9 Conclusions

The cluster‐level GLM with a normal distribution provides a robust alternative to estimating equations with similar power for estimating marginal odds ratios for CRTs. It can be considered both with informative and uninformative cluster size. We recommend the cluster‐level GLM with a normal distribution when the marginal cluster‐average estimands are of interest, and IEE when the marginal individual‐average estimand is of interest.

## Author Contributions

J.A.T. and B.C.K. jointly conceived the idea for the cluster‐level GLM. J.A.T. designed and implemented the simulation study. A.C. wrote the first draft of the Introduction, and B.C.K. wrote the first draft of the Analysis Methods section. J.A.T. wrote the first draft of the remainder of the manuscript. All authors reviewed, contributed to the design of the study, interpretation of the results, revised the manuscript for important intellectual content, and approved the final version of the manuscript.

## Funding

This work was supported by Medical Research Council, MR/X029492/1 and UK Medical Research Council, UKR1934.

## Conflicts of Interest

The authors declare no conflicts of interest.

## Supporting information


**Supplementary Text 1.** Implementation of cluster‐level GLM.
**Supplementary Text 2**. Estimating true effects.
**Supplementary Table 1**. Uninformative cluster size. Mean estimated odds ratios.
**Supplementary Table 2**. Uninformative cluster size. Relative error in estimated standard errors.
**Supplementary Table 3**. Uninformative cluster size. Confidence interval coverage.
**Supplementary Table 4**. Uninformative cluster size. Power.
**Supplementary Table 5**. Informative cluster size. Mean estimated odds ratios.
**Supplementary Table 6**. Informative cluster size. Relative error in estimated standard errors.
**Supplementary Table 7:** Informative cluster size. Confidence interval coverage.
**Supplementary Table 8**. Informative cluster size. Power.

## Data Availability

The data that support the findings of this study are openly available on Github at https://github.com/jenniferthompson1/Cluster_level_GLM.
