# Supplementary material for: Estimating Marginal Odds Ratios for Cluster Randomized Trials: The Cluster‐Level GLM
Source: Stat Med. 2026 Oct 21;45(25-27):e70794. doi: 10.1002/sim.70794 (PMC13687777; doi:10.1002/sim.70794)
Supplement: Supplementary file 1 [file SIM-45-0-s001.pdf]

Supplementary simulation study methods

Supplementary text 1: Implementation of cluster-level GLM

This method requires data summarised to the cluster level, so that there is one row of data per cluster. p is the proportion with the outcome in each cluster, arm contains 0 for control clusters and 1 for intervention clusters, clustersize contains the number of observations in each cluster.

Unweighted Cluster-level GLM with a normal distribution:

```
glm p arm, family(normal) link(logit)
```

Unweighted Cluster-level GLM with a gamma distribution:

```
glm p arm, family(gamma) link(logit)
```

Weighted cluster-level GLM with a normal distribution

```
glm p arm [pw = clustersize], family(normal) link(logit)
```

After each model run the following code to calculate p values and confidence intervals using number of clusters – 2 as the degrees of freedom

```
local df = /// Set the degrees of freedom

local  p = 2 * ttail(`df', abs(_b[arm] / _se[arm]))

local lowci = _b[arm] - invt(`df', 0.975) * _se[arm]

local uppci = _b[arm] + invt(`df', 0.975) * _se[arm]

di “p=`p’ 95% CI (`lowci’ , `uppci’)
```

Supplementary text 2: Estimating true effects

To estimate the true marginal odds ratios, we generated one, large dataset for each scenario. These used the same data generating process and parameters for each scenario but with 100,000 clusters. For each cluster, we generated a number of participants with the outcome under the control condition, and the reset the random number seed to also generate a number of participants with the outcome under the intervention condition. These control and intervention outcomes were then used to calculate each of the true odds ratios required for the simulation study.

Supplementary tables

Supplementary Table 1: Uninformative cluster size. Mean estimated odds ratios

Number of clusters	ICC	CV cluster size	unCL-GLM(normal)	unCL-GLM(gamma)	wIEE	wCL-GLM(normal)	unIEE	unGEE
10	.05	0	0.76 (0.75, 0.77)	0.76 (0.75, 0.77)	0.76 (0.75, 0.77)	0.76 (0.75, 0.77)	0.76 (0.75, 0.77)	0.76 (0.75, 0.77)
10	.05	.5	0.76 (0.75, 0.77)	0.76 (0.75, 0.77)	0.76 (0.75, 0.77)	0.76 (0.75, 0.77)	0.76 (0.75, 0.77)	0.76 (0.75, 0.77)
10	.15	0	0.77 (0.75, 0.78)	0.77 (0.75, 0.78)	0.77 (0.75, 0.78)	0.77 (0.75, 0.78)	0.77 (0.75, 0.78)	0.77 (0.75, 0.78)
10	.15	.5	0.77 (0.75, 0.78)	0.77 (0.75, 0.78)	0.77 (0.75, 0.78)	0.76 (0.75, 0.78)	0.76 (0.75, 0.78)	0.77 (0.75, 0.78)
20	.05	0	0.75 (0.75, 0.76)	0.75 (0.75, 0.76)	0.75 (0.75, 0.76)	0.75 (0.75, 0.76)	0.75 (0.75, 0.76)	0.75 (0.75, 0.76)
20	.05	.5	0.75 (0.75, 0.76)	0.75 (0.75, 0.76)	0.75 (0.75, 0.76)	0.75 (0.75, 0.76)	0.75 (0.75, 0.76)	0.76 (0.75, 0.76)
20	.15	0	0.77 (0.76, 0.78)	0.77 (0.76, 0.78)	0.77 (0.76, 0.78)	0.77 (0.76, 0.78)	0.77 (0.76, 0.78)	0.77 (0.76, 0.78)
20	.15	.5	0.78 (0.77, 0.79)	0.78 (0.77, 0.79)	0.78 (0.77, 0.79)	0.78 (0.77, 0.79)	0.78 (0.77, 0.79)	0.78 (0.77, 0.79)
50	.05	0	0.76 (0.75, 0.76)	0.76 (0.75, 0.76)	0.76 (0.75, 0.76)	0.76 (0.75, 0.76)	0.76 (0.75, 0.76)	0.76 (0.75, 0.76)
50	.05	.5	0.76 (0.76, 0.76)	0.76 (0.76, 0.76)	0.76 (0.76, 0.76)	0.76 (0.75, 0.76)	0.76 (0.75, 0.76)	0.76 (0.76, 0.76)
50	.15	0	0.78 (0.77, 0.78)	0.78 (0.77, 0.78)	0.78 (0.77, 0.78)	0.78 (0.77, 0.78)	0.78 (0.77, 0.78)	0.78 (0.77, 0.78)
50	.15	.5	0.78 (0.77, 0.79)	0.78 (0.77, 0.79)	0.78 (0.77, 0.79)	0.78 (0.77, 0.78)	0.78 (0.77, 0.78)	0.78 (0.77, 0.79)

Supplementary Table 2: Uninformative cluster size. Relative error in estimated standard errors

Number of clusters	ICC	CV cluster size	unCL-GLM(normal)	unCL-GLM(gamma)	wIEE	wCL-GLM(normal)	unIEE	unGEE
10	.05	0	1.4 (-2.1, 5.0)	2.9 (-0.7, 6.5)	6.8 (3.0, 10.5)	-4.4 (-7.8, -1.1)	6.8 (3.0, 10.5)	6.8 (3.0, 10.5)
10	.05	.5	-1.2 (-4.7, 2.3)	0.4 (-3.1, 4.0)	20.0 (15.6, 24.4)	-11.0 (-14.2, -7.8)	5.4 (1.6, 9.2)	3.0 (-0.6, 6.7)
10	.15	0	0.3 (-3.2, 3.8)	3.1 (-0.5, 6.7)	5.4 (1.7, 9.0)	-5.7 (-9.0, -2.4)	5.4 (1.7, 9.0)	5.4 (1.7, 9.0)
10	.15	.5	-1.0 (-4.4, 2.5)	2.0 (-1.6, 5.6)	21.8 (17.4, 26.3)	-13.3 (-16.4, -10.3)	2.8 (-0.9, 6.5)	3.1 (-0.5, 6.7)
20	.05	0	-0.1 (-3.5, 3.3)	1.2 (-2.3, 4.6)	2.4 (-1.1, 5.9)	-2.8 (-6.1, 0.5)	2.4 (-1.1, 5.9)	2.4 (-1.1, 5.9)
20	.05	.5	-0.0 (-3.4, 3.4)	1.3 (-2.1, 4.8)	12.7 (8.8, 16.7)	-5.6 (-8.9, -2.4)	2.5 (-1.0, 6.1)	1.8 (-1.7, 5.2)
20	.15	0	2.0 (-1.4, 5.5)	3.8 (0.3, 7.3)	4.6 (1.0, 8.1)	-0.8 (-4.1, 2.6)	4.6 (1.0, 8.1)	4.6 (1.0, 8.1)
20	.15	.5	1.5 (-1.9, 5.0)	3.3 (-0.2, 6.8)	18.3 (14.2, 22.4)	-4.3 (-7.6, -1.0)	4.3 (0.7, 7.9)	3.7 (0.2, 7.2)
50	.05	0	0.3 (-3.1, 3.7)	1.3 (-2.1, 4.7)	1.3 (-2.1, 4.7)	-0.8 (-4.1, 2.6)	1.3 (-2.1, 4.7)	1.3 (-2.1, 4.7)
50	.05	.5	0.2 (-3.2, 3.5)	1.2 (-2.2, 4.6)	9.2 (5.5, 12.9)	-1.4 (-4.7, 2.0)	2.0 (-1.4, 5.5)	1.1 (-2.3, 4.5)
50	.15	0	-1.5 (-4.8, 1.8)	-0.5 (-3.8, 2.9)	-0.6 (-3.9, 2.8)	-2.6 (-5.8, 0.7)	-0.6 (-3.9, 2.8)	-0.6 (-3.9, 2.8)
50	.15	.5	-0.3 (-3.6, 3.0)	0.8 (-2.5, 4.2)	11.8 (8.0, 15.6)	-3.3 (-6.6, -0.1)	0.0 (-3.3, 3.4)	0.4 (-3.0, 3.8)

Supplementary Table 3: Uninformative cluster size. Confidence interval coverage

Number of clusters	ICC	CV cluster size	unCL-GLM(normal)	unCL-GLM(gamma)	wIEE	wCL-GLM(normal)	unIEE	unGEE
10	.05	0	95.3 (94.3, 96.3)	95.7 (94.7, 96.6)	96.2 (95.3, 97.1)	94.2 (93.1, 95.3)	96.2 (95.3, 97.1)	96.2 (95.3, 97.1)
10	.05	.5	94.5 (93.5, 95.6)	95.1 (94.1, 96.1)	96.2 (95.3, 97.1)	91.2 (89.9, 92.5)	94.4 (93.4, 95.5)	94.6 (93.5, 95.6)
10	.15	0	95.4 (94.5, 96.4)	96.4 (95.5, 97.2)	96.1 (95.2, 97.0)	94.2 (93.1, 95.3)	96.1 (95.2, 97.0)	96.1 (95.2, 97.0)
10	.15	.5	95.3 (94.3, 96.3)	96.4 (95.5, 97.2)	96.9 (96.0, 97.7)	91.7 (90.4, 93.0)	94.6 (93.5, 95.6)	95.7 (94.7, 96.6)
20	.05	0	95.2 (94.2, 96.2)	95.5 (94.5, 96.4)	95.5 (94.5, 96.5)	94.6 (93.6, 95.7)	95.5 (94.5, 96.5)	95.5 (94.5, 96.5)
20	.05	.5	94.9 (93.9, 96.0)	95.6 (94.6, 96.5)	96.3 (95.4, 97.2)	93.0 (91.8, 94.2)	94.8 (93.8, 95.8)	95.2 (94.2, 96.2)
20	.15	0	95.3 (94.3, 96.3)	96.1 (95.2, 97.0)	95.7 (94.8, 96.7)	94.6 (93.5, 95.7)	95.7 (94.8, 96.7)	95.7 (94.8, 96.7)
20	.15	.5	95.8 (94.8, 96.7)	96.4 (95.6, 97.3)	97.3 (96.6, 98.1)	94.1 (93.0, 95.2)	95.5 (94.5, 96.4)	96.2 (95.3, 97.1)
50	.05	0	95.2 (94.2, 96.2)	95.6 (94.6, 96.5)	95.5 (94.5, 96.4)	95.0 (94.0, 96.0)	95.5 (94.5, 96.4)	95.5 (94.5, 96.4)
50	.05	.5	94.8 (93.8, 95.9)	95.2 (94.2, 96.2)	96.2 (95.3, 97.1)	93.9 (92.8, 95.1)	94.8 (93.8, 95.9)	95.1 (94.1, 96.1)
50	.15	0	94.6 (93.6, 95.7)	95.3 (94.3, 96.3)	94.7 (93.7, 95.8)	94.5 (93.4, 95.5)	94.7 (93.7, 95.8)	94.7 (93.7, 95.8)
50	.15	.5	94.8 (93.8, 95.9)	95.2 (94.2, 96.2)	96.3 (95.4, 97.2)	94.1 (93.0, 95.2)	94.8 (93.7, 95.8)	95.2 (94.2, 96.2)

Supplementary Table 4: Uninformative cluster size. Power

Number of clusters	ICC	CV cluster size	unCL-GLM(normal)	unCL-GLM(gamma)	wIEE	wCL-GLM(normal)	unIEE	unGEE
10	.05	0	13.8 (12.1, 15.4)	12.2 (10.7, 13.7)	11.9 (10.4, 13.4)	16.3 (14.5, 18.0)	11.9 (10.4, 13.4)	11.9 (10.4, 13.4)
10	.05	.5	13.7 (12.1, 15.3)	12.6 (11.0, 14.1)	10.1 (8.7, 11.5)	18.6 (16.8, 20.5)	13.4 (11.8, 15.0)	12.7 (11.2, 14.3)
10	.15	0	7.9 (6.6, 9.1)	5.8 (4.7, 6.9)	6.8 (5.6, 8.0)	9.6 (8.2, 11.0)	6.8 (5.6, 8.0)	6.8 (5.6, 8.0)
10	.15	.5	7.9 (6.7, 9.2)	6.0 (4.9, 7.1)	5.4 (4.4, 6.5)	11.6 (10.1, 13.1)	7.7 (6.5, 9.0)	7.2 (6.0, 8.5)
20	.05	0	27.2 (25.1, 29.3)	26.3 (24.2, 28.3)	25.7 (23.6, 27.7)	29.0 (26.9, 31.2)	25.7 (23.6, 27.7)	25.7 (23.6, 27.7)
20	.05	.5	25.9 (23.8, 27.9)	24.8 (22.8, 26.8)	21.0 (19.1, 22.9)	27.9 (25.8, 30.0)	23.8 (21.8, 25.8)	25.1 (23.1, 27.2)
20	.15	0	11.0 (9.5, 12.5)	9.7 (8.3, 11.1)	10.4 (9.0, 11.9)	12.5 (11.0, 14.1)	10.4 (9.0, 11.9)	10.4 (9.0, 11.9)
20	.15	.5	10.5 (9.1, 11.9)	9.0 (7.7, 10.3)	7.4 (6.1, 8.6)	12.4 (10.8, 13.9)	9.3 (8.0, 10.7)	9.7 (8.3, 11.1)
50	.05	0	59.3 (57.0, 61.6)	58.8 (56.5, 61.1)	58.6 (56.3, 60.9)	59.9 (57.6, 62.2)	58.6 (56.3, 60.9)	58.6 (56.3, 60.9)
50	.05	.5	56.0 (53.6, 58.3)	55.5 (53.2, 57.8)	49.3 (46.9, 51.6)	53.4 (51.1, 55.8)	50.7 (48.4, 53.1)	55.9 (53.6, 58.3)
50	.15	0	23.3 (21.3, 25.3)	22.4 (20.4, 24.3)	22.7 (20.7, 24.7)	23.9 (21.9, 25.9)	22.7 (20.7, 24.7)	22.7 (20.7, 24.7)
50	.15	.5	22.6 (20.6, 24.5)	21.6 (19.6, 23.5)	17.3 (15.5, 19.1)	23.0 (21.1, 25.0)	21.1 (19.2, 23.1)	22.3 (20.4, 24.3)

Supplementary Table 5: Informative cluster size. Mean estimated odds ratios

Number of clusters	ICC	True marginal cluster-average odds ratio	unCL-GLM(normal)	wIEE	True marginal participant-average odds ratio	wCL-GLM(normal)	unIEE	unGEE
10	.05	0.89	0.89 (0.87, 0.90)	0.89 (0.87, 0.90)	0.84	0.84 (0.83, 0.86)	0.84 (0.83, 0.86)	0.92 (0.91, 0.93)
20	.05	0.89	0.89 (0.88, 0.90)	0.89 (0.88, 0.90)	0.84	0.85 (0.84, 0.86)	0.85 (0.84, 0.86)	0.92 (0.91, 0.93)
50	.05	0.89	0.89 (0.88, 0.89)	0.89 (0.88, 0.89)	0.84	0.84 (0.84, 0.85)	0.84 (0.84, 0.85)	0.93 (0.92, 0.93)
10	.15	0.90	0.89 (0.87, 0.91)	0.89 (0.87, 0.91)	0.85	0.86 (0.84, 0.88)	0.86 (0.84, 0.88)	0.93 (0.90, 0.95)
20	.15	0.90	0.89 (0.88, 0.90)	0.89 (0.88, 0.90)	0.85	0.85 (0.83, 0.86)	0.85 (0.83, 0.86)	0.93 (0.91, 0.95)
50	.15	0.90	0.90 (0.89, 0.90)	0.90 (0.89, 0.90)	0.85	0.85 (0.84, 0.86)	0.85 (0.84, 0.86)	0.94 (0.93, 0.95)

Supplementary Table 6: Informative cluster size. Relative error in estimated standard errors

Number of clusters	ICC	unCL-GLM(normal)	wIEE	wCL-GLM(normal)	unIEE	unGEE
10	.05	-1.5 (-5.0, 2.0)	20.9 (16.3, 25.4)	-12.9 (-16.0, -9.8)	3.5 (-0.3, 7.3)	0.8 (-5.1, 6.7)
20	.05	-0.1 (-3.5, 3.3)	14.4 (10.4, 18.4)	-6.3 (-9.5, -3.0)	2.0 (-1.5, 5.6)	-7.8 (-11.0, -4.6)
50	.05	-0.1 (-3.5, 3.2)	9.9 (6.1, 13.6)	-1.7 (-5.0, 1.6)	1.7 (-1.7, 5.2)	-13.7 (-16.6, -10.8)
10	.15	2.0 (-1.5, 5.6)	26.4 (21.8, 31.1)	-10.6 (-13.7, -7.4)	6.4 (2.6, 10.3)	0.3 (-4.1, 4.7)
20	.15	-0.9 (-4.3, 2.5)	15.2 (11.3, 19.2)	-6.3 (-9.6, -3.1)	2.1 (-1.4, 5.6)	-9.5 (-12.6, -6.5)
50	.15	2.4 (-1.1, 5.8)	15.0 (11.1, 18.9)	0.1 (-3.2, 3.5)	3.6 (0.1, 7.1)	-12.9 (-15.8, -10.0)

Supplementary Table 7: Informative cluster size. Confidence interval coverage

Number of clusters	ICC	unCL-GLM(normal)	wIEE	wCL-GLM(normal)	unIEE	unGEE
10	.05	95.1 (94.0, 96.1)	96.7 (95.9, 97.5)	91.9 (90.6, 93.2)	94.8 (93.8, 95.8)	95.9 (94.9, 96.8)
20	.05	94.9 (93.9, 95.9)	96.2 (95.3, 97.1)	93.0 (91.8, 94.2)	94.5 (93.4, 95.6)	92.7 (91.4, 93.9)
50	.05	94.7 (93.6, 95.7)	96.1 (95.2, 97.0)	93.9 (92.8, 95.0)	94.6 (93.5, 95.7)	85.6 (83.9, 87.2)
10	.15	95.8 (94.9, 96.7)	97.2 (96.5, 98.0)	92.3 (91.0, 93.6)	95.1 (94.1, 96.1)	96.4 (95.6, 97.3)
20	.15	94.8 (93.7, 95.8)	96.6 (95.7, 97.5)	93.0 (91.8, 94.2)	94.8 (93.8, 95.8)	92.7 (91.5, 94.0)
50	.15	95.6 (94.6, 96.6)	97.5 (96.7, 98.2)	94.8 (93.8, 95.8)	95.2 (94.2, 96.2)	90.4 (89.1, 91.8)

Supplementary Table 8: Informative cluster size. Power

Number of clusters	ICC	unCL-GLM(normal)	wIEE	wCL-GLM(normal)	unIEE	unGEE
10	.05	6.6 (5.5, 7.8)	4.6 (3.6, 5.6)	11.5 (10.0, 13.0)	7.1 (5.9, 8.3)	5.0 (4.0, 6.0)
20	.05	9.0 (7.7, 10.4)	6.8 (5.6, 8.0)	15.1 (13.4, 16.8)	12.2 (10.7, 13.7)	8.1 (6.8, 9.4)
50	.05	14.5 (12.8, 16.1)	11.1 (9.6, 12.6)	23.1 (21.2, 25.1)	21.6 (19.7, 23.5)	12.9 (11.3, 14.4)
10	.15	4.9 (3.9, 6.0)	3.2 (2.4, 4.0)	8.2 (6.9, 9.5)	5.4 (4.3, 6.4)	4.1 (3.2, 5.1)
20	.15	6.3 (5.1, 7.4)	4.2 (3.3, 5.1)	9.6 (8.2, 10.9)	7.1 (5.9, 8.3)	7.3 (6.1, 8.5)
50	.15	7.3 (6.1, 8.6)	5.0 (3.9, 6.0)	11.5 (10.0, 13.0)	10.4 (8.9, 11.8)	9.0 (7.7, 10.3)
